# On the female of *Gypona reversa* DeLong & Martinson, 1972, with emphasis on genital structures (Insecta: Hemiptera: Cicadellidae)

**DOI:** 10.3897/BDJ.2.e4272

**Published:** 2014-12-31

**Authors:** Elidiomar R. Da-Silva, Luci B. N. Coelho, Paulo Sérgio F. Ferreira

**Affiliations:** † Universidade Federal do Estado do Rio de Janeiro, Rio de Janeiro, Brazil; ‡ Universidade Federal de Viçosa, Viçosa, Brazil

**Keywords:** Taxonomy, Morphology, Female genitalia, Leafhopper, Gyponini, Neotropics

## Abstract

*Gypona reversa* DeLong & Martinson, 1972 has its ovipositor described and illustrated based on the examination of specimens from its type locality. This is the first species of *Gypona* Germar, 1821 to have the female genitalia detailed description published.

## Introduction

The Pan-American genus *Gypona* Germar, 1821 (Cicadellidae: Iassinae: Gyponini) includes about 200 described species (DeLong 1980). Most of the species are only described based on the male, and when the female is known there is only reference to the sternite VII as the genital structures (Coelho et al. 2001, Azevedo-Filho and Carvalho 2003). *Gypona (G.) reversa* DeLong & Martinson, 1972 was described based on male holotype (from Viçosa, Minas Gerais, Brasil) and paratype (from Jalapa, Mexico) (Coelho et al. 2001). Coelho et al. (2001) redescribed the male and described for the first time the female based on specimens from Viçosa. As for the female genitalia, only the sternite VII was described. In view of recent studies that emphasize the importance of female genital structures in Cicadellidae (e.g., Rodriguez and Mejdalani 2009, Mejdalani and Silva 2010, Mejdalani and Cavichioli 2013, Domahovski et al. 2014), we herein give a detailed redescription of the female of *G. reversa*.

## Materials and methods

The descriptive terminology adopted herein follows mainly Young (1977), except for the female terminalia (Balduf 1934, Blocker and Triplehorn 1985). For morphological study of the genitalia, abdomen was removed and soaked it in a warm solution of 10% KOH, rinsed in water and stored in glycerin (modified from Oman 1949). Photographs were taken with a digital camera EC3 attached to a stereomicroscope Leica S8AP0, and a camera DMC 2900 attached to a microscope Leica DM4000 B LED, using the image stacking software CombineZP (www.hadleyweb.pwp.blueyonder.co.uk). Terminalia were stored in a small vial with glycerin pinned below the specimen.

The specimen was collected in a fragment of Atlantic Forest inserted on the Universidade Federal de Viçosa (UFV) campus. UFV is located in Viçosa municipality, “Zona da Mata” of Minas Gerais State, southeastern Brazil (Rocha et al. 2007) and has an area of 1,359 hectares (Brianezi et al. 2013). The climate is Cwa (Köppen), mesothermal with hot rainy summers, cool dry winters, average annual temperature 21.8°C, and average annual rainfall 1314.2 mm (Castro et al. 1983, Brianezi et al. 2013).

The specimen examined belongs to Coleção Entomológica José Alfredo Pinheiro Dutra, Departamento de Zoologia (DZRJ), Instituto de Biologia, Universidade Federal do Rio de Janeiro, Rio de Janeiro, Brazil.

## Taxon treatments

### Gypona (Gypona) reversa

DeLong & Martinson, 1972

#### Materials

- **Type status:** Other material. **Occurrence:** recordedBy: Luci B. N. Coelho; individualCount: 1; sex: female; lifeStage: adult; **Taxon:** taxonID: Native; scientificNameID: lsid:zoobank.org:act:8B3CBB30-41F7-4CE3-ADD5-8C00FE4D4D6A; acceptedNameUsage: Gypona reversa DeLong & Martinson, 1972; kingdom: Animalia; phylum: Arthropoda; class: Hexapoda; order: Hemiptera; family: Cicadellidae; genus: Gypona; subgenus: Gypona; specificEpithet: reversa; scientificNameAuthorship: DeLong & Martinson, 1972; **Location:** continent: South America; country: Brazil; countryCode: BR; stateProvince: Minas Gerais; municipality: Viçosa; locality: Mata da Biologia, Recanto das Cigarras; verbatimElevation: 650 m; verbatimCoordinates: 20°45'31.8"S 42°51'40.5"W; **Identification:** identifiedBy: Luci B. N. Coelho; dateIdentified: 1993; **Event:** eventDate: 08/09/1993; **Record Level:** language: en; institutionCode: DZRJ; collectionCode: Insects; ownerInstitutionCode: Coleção Entomológica Professor José Alfredo Pinheiro Dutra; basisOfRecord: PreservedSpecimen
- **Type status:** Other material. **Occurrence:** recordedBy: Luci B. N. Coelho; individualCount: 1; sex: female; lifeStage: adult; **Taxon:** taxonID: Native; scientificNameID: lsid:zoobank.org:act:8B3CBB30-41F7-4CE3-ADD5-8C00FE4D4D6A; acceptedNameUsage: Gypona reversa DeLong & Martinson, 1973; kingdom: Animalia; phylum: Arthropoda; class: Hexapoda; order: Hemiptera; family: Cicadellidae; genus: Gypona; subgenus: Gypona; specificEpithet: reversa; **Location:** continent: South America; country: Brazil; countryCode: BR; stateProvince: Minas Gerais; municipality: Viçosa; locality: Mata da Biologia, Recanto das Cigarras; verbatimElevation: 650 m; verbatimCoordinates: 20°45'31.8"S 42°51'40.5"W; **Identification:** identifiedBy: Luci B. N. Coelho; dateIdentified: 1993; **Event:** eventDate: 06/28/1993; **Record Level:** language: en; institutionCode: DZRJ; collectionCode: Insects; ownerInstitutionCode: Coleção Entomológica Professor José Alfredo Pinheiro Dutra; basisOfRecord: PreservedSpecimen

#### Description

***Gypona reversa* DeLong & Martinson, 1972**

**Redescription of female**

**Figure 1.**
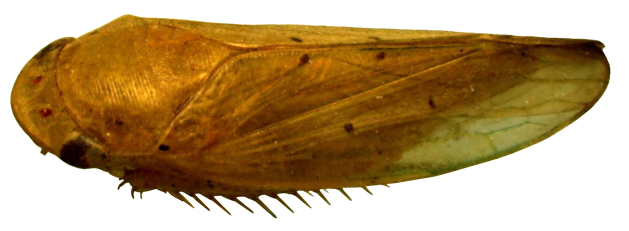
*Gypona reversa* DeLong & Martinson, 1972, female, dorso-lateral view.

Length 7.8 mm. General color green with black and brown spots on pronotum and wings (Figs 1, 2a). Crown transversely striated, anterior margin rounded; transocular width 4.5 times median length, inter-ocular width 3.0 times median length; eyes brown, ocelli red; transocular width about 0.9 times pronotum maximum width (Fig. 2a). Face greenish-yellow, clypeus length about 1.5 times width, apical margin slightly indented medially (Fig. 2b). Pronotum transversely striated, black spot behind each eye; maximum width about twice median length. Scutellum transversely striated (Fig. 2a).

Forewings translucent, apical region smoky, black spot at the insertion point; brown spot at the apex of each claval vein, inner discal cell with two brown spots, one basal and other apical; irregular brown band from clavus apex to median portion of fifth apical cell; about 3.3 times longer than wide; appendix brown, vestigial.

Sternite VII (Fig. 3a, c) 2.4 times longer than preceding segment; median area, anterolateral angles and posterior margin, brown; lateral margin 1.2 times median length, margin convex, posterolateral corners rounded; posterior margin slightly elevated, median third with two "teeth" separated by shallow concavity, lateral third slightly concave (Coelho et al. 2001); maximum width about 2.4 median length.

**Figure 8.**
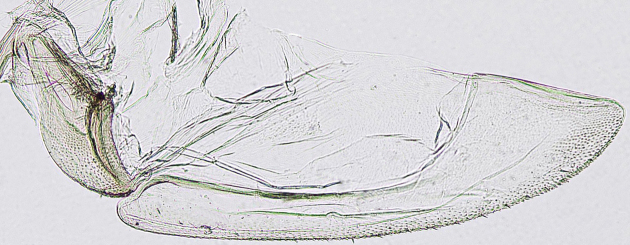
*Gypona reversa* DeLong & Martinson, 1972, female, valvifer II and valvula III, lateral view.

Pygofer, in lateral view, approximately triangular shaped, with apex rounded; scattered macrosetae at posterior margin (Fig. 3d). Ovipositor much shorter than pygofer. Ovipositor, in ventral view, with a median lateral undulation (Fig. 3a), which actually represents the lateral undulation of valvulae II (Fig. 3b). First valvifer approximately quadrangular (Fig. 4a). Valvula I (Fig. 4), in lateral view, about 4.5 times longer than broad; apex acute, distinctly narrowed at tip (dorsal margin incised); dorsal sculptured area finely strigate (Fig. 5a); ventral fold undeveloped (Figs 4, 5b), apical ventral area strigate, subapical ventral area sculptured with longitudinal striae (Figs 4b, 5b); ramus extending to apical end; base of valvula extending anteriad of 1st valvifer. Right and left valvulae II similar in shape and size, strongly fused to each other, and expanded apically; approximately 4 times longer than broader (Fig. 6a); with broadest point on apical half; ventral margin of apical half, rounded (Fig. 6a, b), dorsoapical margin straight, finely serrated (Figs 6, 7a),channels and pores conspicuous (Figs 6c, d, 7b); median-dorsal margin distinctly granular (Figs 6b, 7a); median-lateral undulation in ventral view (Fig. 3b). Second valvifer (Fig. 8) approximately semi-ovalar, point of articulation dark. Valvula III (Fig. 8), in lateral view, approximately 3.44x longer than broad, slightly more sclerotized in ventral margin; ventral half with rows of small conical apical-pointed thorns and scattered spiniform bristles (Fig. 9); ventral margin with apex curved dorsally, dorsal margin more rectilinear (Fig. 8); apex rounded (Figs 8, 9b).

#### Diagnosis

General color green with black and brown spots on pronotum and wings (Figs 1, 2a). Sternite VII (Fig. 3c) with lateral margin convex, posterolateral corners rounded; posterior margin slightly elevated, median third with two "teeth" separated by shallow concavity, lateral third slightly concave. Ovipositor, in ventral view, with a median lateral undulation (Fig. 3a, b), which represents the lateral undulation of valvulae II. Valvula I (Fig. 4) with apex acute, distinctly narrowed at tip; dorsal sculptured area finely strigate, subapical margin with longitudinal striae; ventral fold undeveloped. Valvula II (Figs 6, 7a) with broadest point on apical half; dorsoapical margin truncate finely serrated; median-dorsal margin distinctly granular. Valvula III (Figs 8, 9) with ventral half with rows of small conical apical-pointed thorns and scattered spiniform bristles; ventral margin with apex curved dorsally, dorsal margin more rectilinear.

#### Distribution

Brazil (Minas Gerais State, Viçosa municipality) and Mexico (Veracruz State, Xalapa municipality - formerly known as "Jalapa") (DeLong and Martinson 1972, Coelho et al. 2001).

#### Biology

Specimens were collected in a grove of Atlantic forest, feeding on *Wedelia paludosa* DC (Compositae).

#### Notes

**Studied specimens**

BRAZIL, Minas Gerais, Viçosa, Universidade Federal de Viçosa, Recanto das Cigarras, 20°45'31.8"S 42°51'40.5"W (in *Wedelia paludosa* DC, Compositae), L.B.N. Coelho leg., 1 ♀, 27/vi/1993; 1 ♀, 09/viii/1993 (DZRJ).

## Discussion

This is the first published description of the ovipositor valvulae of any *Gypona* species. Hill (1970), in his unpublished thesis, illustrated the valvula II of *Gypona verticalis* Stål, 1864, which is quite shorter than in *G. reversa* (Fig. 6a). Noronha (2000), in her unpublished monograph, briefly described the female genitalia of *G. hiata* DeLong and Freytag, 1967. Compared with *G. hiata*, the sternite VII of *G. reversa* has a more convex lateral margin, and posterior margin with two "teeth" separated by shallow concavity (Fig. 3a, c); pygofer is longer (Fig. 3a, d); ventral fold of valvula I is vestigial (Figs 4, 5b); apical third of valvula II is broader, and dorsally serrated, not bearing tooth (Fig. 6c, d); apical third of valvula III is broader (Fig. 8).

According to Engel and Takiya (2012), diagnostic characteristics in the female genitalia often used in other leafhopper groups were found to vary intraspecifically within genus *Clinonana* Osborn, 1988. Nevertheless, the authors believe that researchers should always make an effort to describe the female genitalia in as much detail as possible, so hopefully in the future it might become a source of useful taxonomic characteristics.

## Supplementary Material

XML Treatment for Gypona (Gypona) reversa

## Figures and Tables

**Figure 2a.**
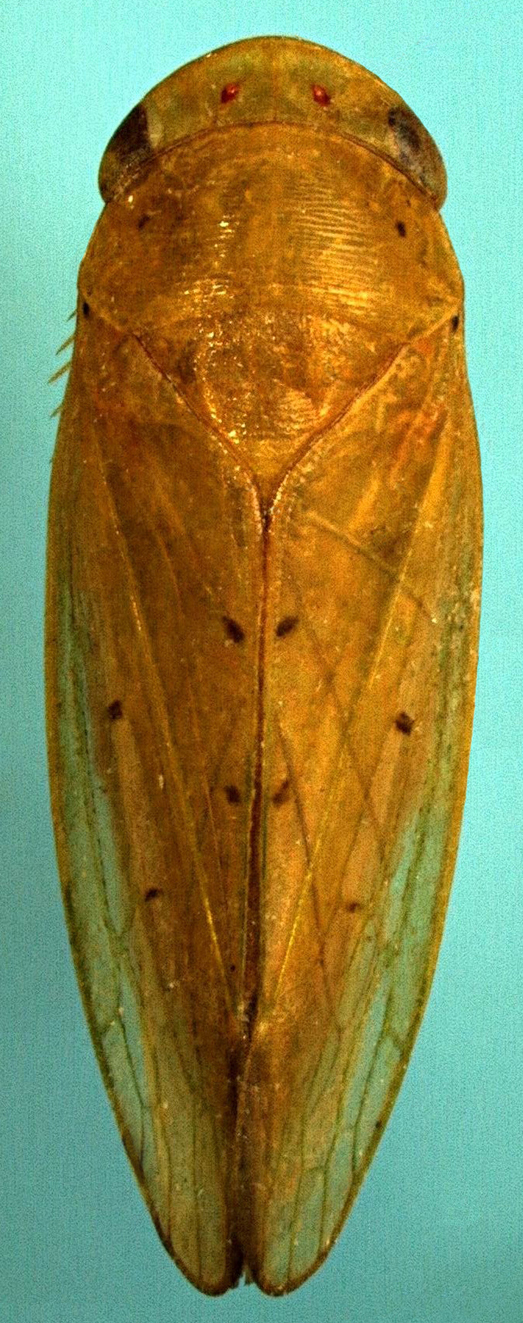
Dorsal view.

**Figure 2b.**
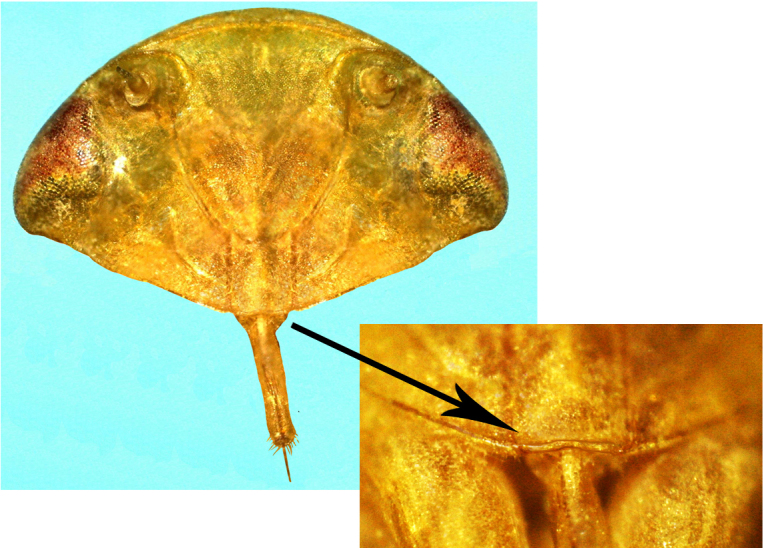
Face (detail: apical margin of clypeus).

**Figure 3a.**
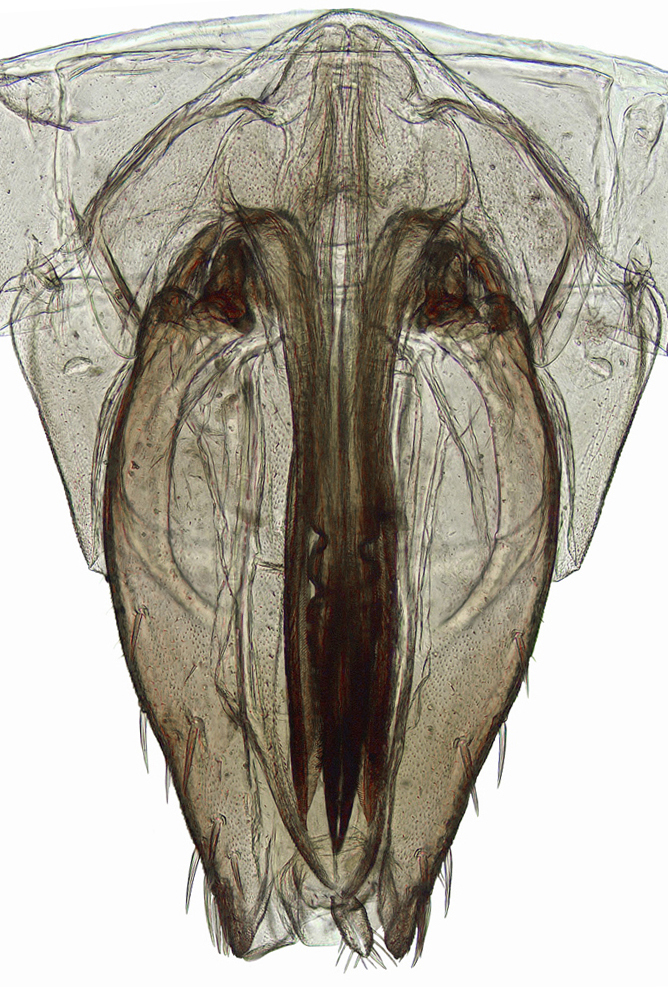
Abdominal tip, ventral view.

**Figure 3b.**
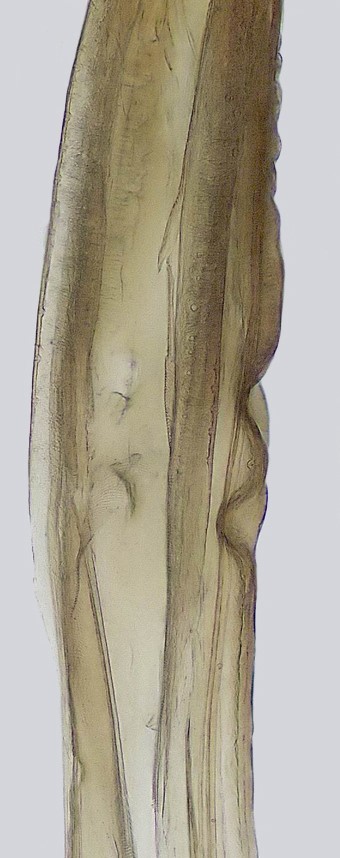
Median lateral undulation of ovipositor valvulae II, ventral view.

**Figure 3c.**
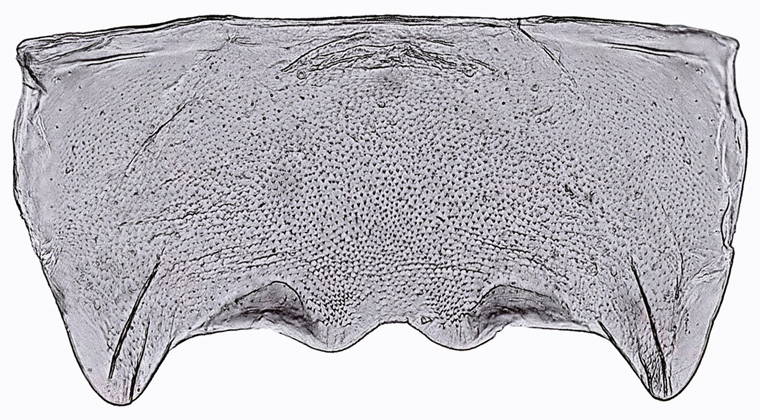
Sternite VII, ventral view.

**Figure 3d.**
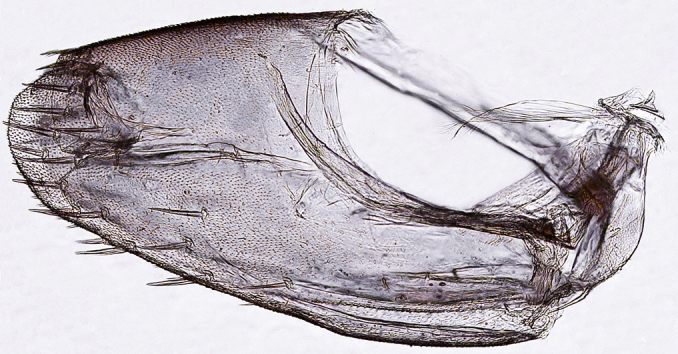
Pygofer, lateral view.

**Figure 4a.**
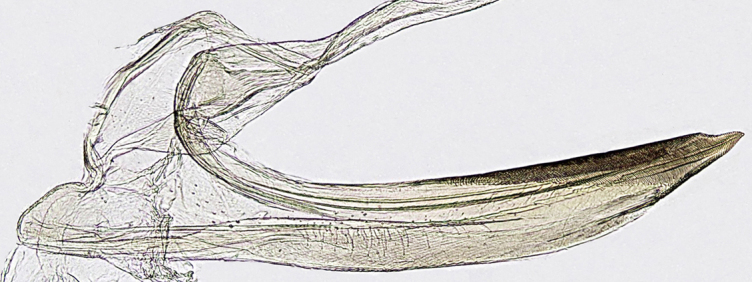
First valvifer and valvula I, lateral view.

**Figure 4b.**
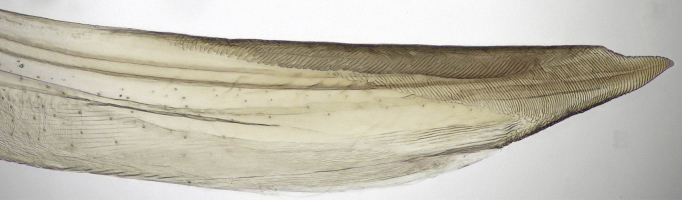
Distal two thirds, lateral view.

**Figure 5a.**
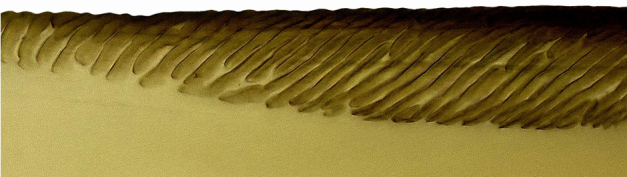
Dorsal strigate area, lateral view.

**Figure 5b.**
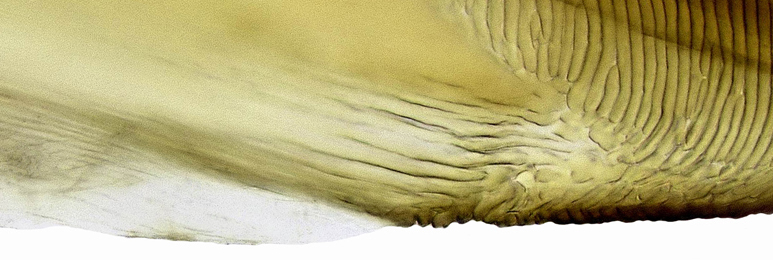
Subapical ventral area, at the limit between longitudinal striae and strigate area, lateral view.

**Figure 6a.**
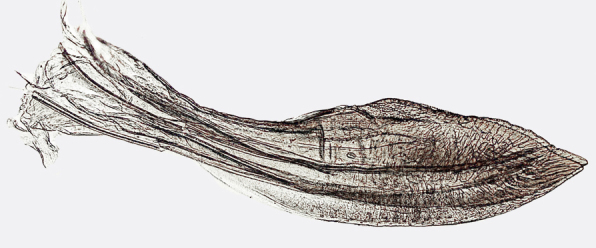
Lateral view.

**Figure 6b.**
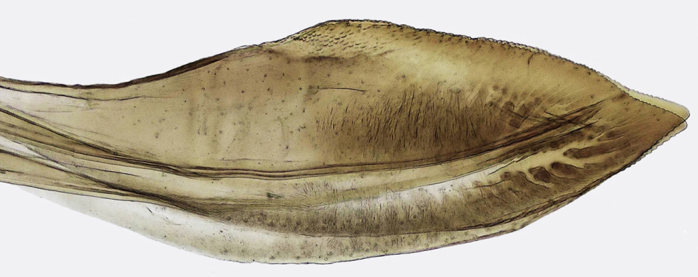
Distal two thirds, lateral view.

**Figure 6c.**
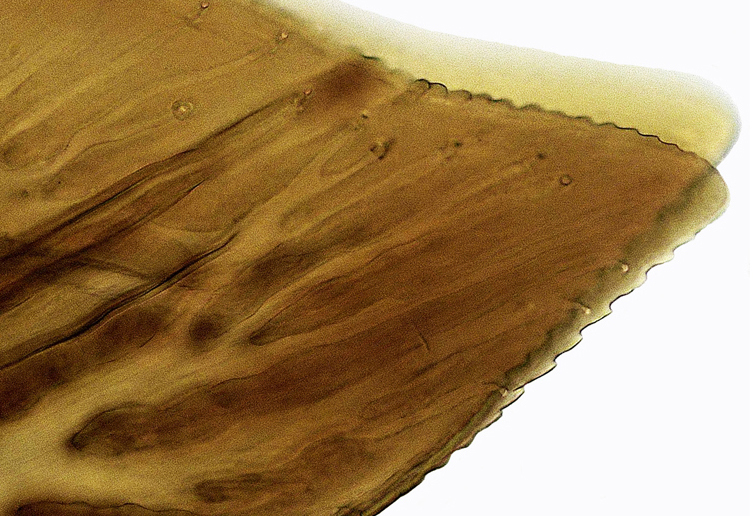
Apical area of left valvula, lateral view.

**Figure 6d.**
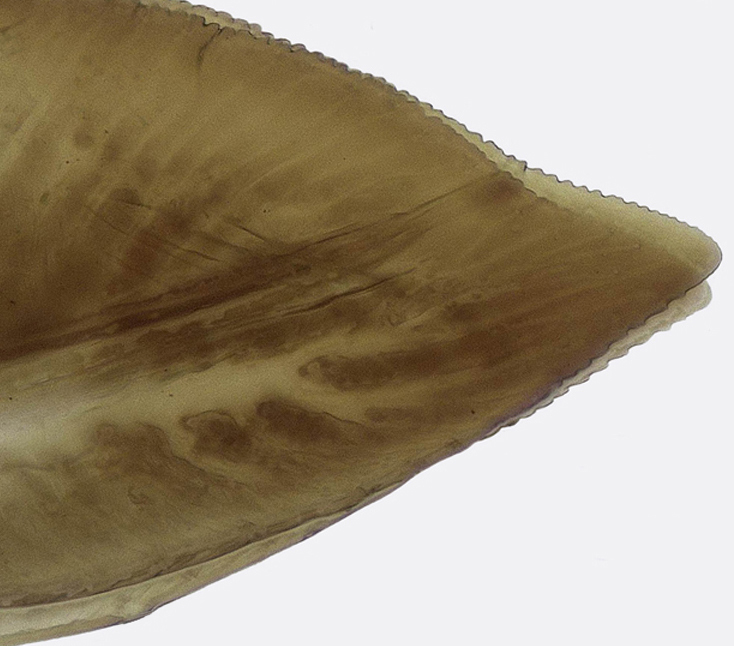
Apical area of right valvula, lateral view.

**Figure 7a.**
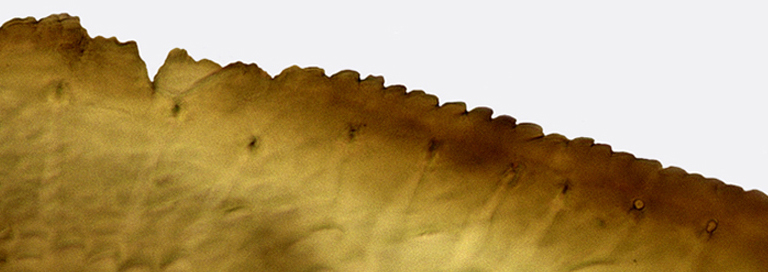
Median-dorsal margin, lateral view.

**Figure 7b.**
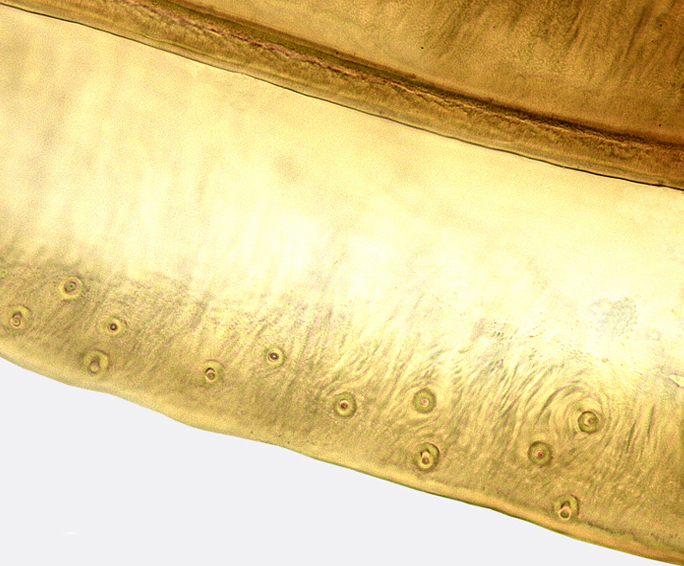
Sub-basal ventral margin, lateral view.

**Figure 9a.**
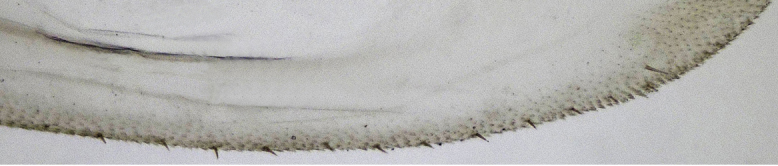
Sub-apical ventral margin, lateral view.

**Figure 9b.**
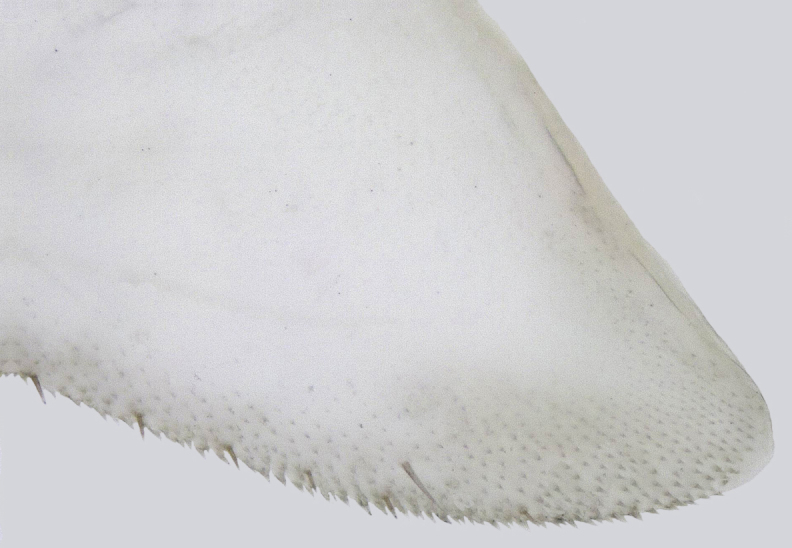
Apical area, ventral view.
